# Effects of Curcuminoids Plus Piperine Co‐Supplementation on Liver Enzymes and Inflammation in Adults: A GRADE‐Assessed Systematic Review and Meta‐Analysis

**DOI:** 10.1002/fsn3.70588

**Published:** 2025-07-14

**Authors:** Mehdi Karimi, Meysam Javadi, Maryam Sharifi, Fereshteh Valizadeh, Mohammad Amin Karimi, Omid Asbaghi

**Affiliations:** ^1^ Faculty of Medicine Bogomolets National Medical University (NMU) Kyiv Ukraine; ^2^ Department of Sport Management, Faculty of Humanities Science University of Science and Culture Tehran Iran; ^3^ Student Research Committee, School of Nutritional Sciences and Food Technology Kermanshah University of Medical Sciences Kermanshah Iran; ^4^ Faculty of Medicine Shahid Beheshti University of Medical Sciences (SBUMS) Tehran Iran; ^5^ Cancer Research Center Shahid Beheshti University of Medical Sciences (SBUMS) Tehran Iran

**Keywords:** curcumin, cytokines, herbal medicine, inflammation, liver, phytochemicals, piperine, traditional Persian medicine

## Abstract

Chronic diseases and inflammation are often linked to elevated liver enzymes and inflammatory markers. Studies suggest that curcuminoid and piperine co‐supplementation (Cur + Pip), which enhances bioavailability, may offer beneficial effects. However, previous findings have been inconsistent. This meta‐analysis aims to evaluate the effectiveness of Cur + Pip on liver enzymes and inflammation in adults. A comprehensive search of scientific databases was conducted until April 2025 to identify relevant randomized controlled trials (RCTs) involving adults. Included studies compared Cur + Pip with placebo, control, or no treatment, focusing on changes in liver enzymes and inflammatory markers as main outcomes. Data from eligible studies were extracted, and pooled effect sizes were calculated using weighted mean differences (WMDs) with 95% confidence intervals (CIs) under a random‐effects model. The pooled analysis of 18 RCTs showed that Cur + Pip significantly reduced AST (WMD: −2.29 IU, *p* = 0.019) and IL‐6 (WMD: −5.59, *p* < 0.001), whereas changes in ALT (WMD: −3.88 IU, *p* = 0.053), ALP (WMD: −15.74 IU, *p* = 0.095), CRP (WMD: −1.53 mg/dL, *p* = 0.222), and TNF‐α (WMD: −0.77, *p* = 0.314) were not statistically significant. Subgroup analysis revealed that reductions in ALT were significant in individuals with metabolic dysfunction (WMD: −4.64 IU, *p* = 0.044) and adults with overweight (WMD: −4.89 IU, *p* = 0.048). AST reductions were more pronounced in prolonged supplementation (≥ 12 weeks) (WMD: −4.08 IU, *p* < 0.001) and in overweight participants (WMD: −3.04 IU, *p* = 0.020). Cur + Pip demonstrated beneficial effects on liver enzymes, particularly AST, and showed a positive trend for ALT, especially in overweight or metabolically at‐risk individuals. The observed reduction in IL‐6 supports their potential anti‐inflammatory properties, although no significant effects were seen for CRP or TNF‐α. These findings suggest therapeutic promise in specific populations, but further large‐scale, long‐duration trials are necessary to confirm efficacy and establish clinical significance.

## Graphical abstract

Curcuminoid and piperine co‐supplementation significantly reduced AST levels but showed no significant effect on ALT or ALP. It also led to a significant decrease in IL‐6, with no significant changes in CRP or TNF‐α levels.

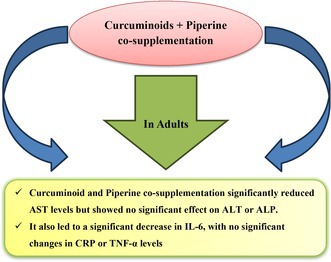

**Abbreviations**
ALP: alkaline phosphatase
ALT: alanine transaminase
AST: aspartate aminotransferase
CRP: C‐reactive protein
IL‐6: interleukin‐6
RCTs: randomized controlled trials
TNF‐α: tumor necrosis factor‐alpha

## 1 Introduction

Liver diseases and chronic inflammation are significant global health challenges, often characterized by elevated liver enzymes, along with increased inflammatory markers (Duan et al. 2022; Gan et al. 2025). These biochemical indicators reflect liver dysfunction and systemic inflammation, both of which contribute to the progression of metabolic disorders, non‐alcoholic fatty liver disease (NAFLD), and other hepatic conditions (Duan et al. 2022; Rinaldi et al. 2021). As a result, identifying effective and natural interventions to reduce liver enzyme levels and modulate inflammatory responses remains a critical area of research. Recent studies have shown that oral supplementation has beneficial effects on metabolic factors (Karimi et al. 2025; Karim et al. 2024).

A substantial body of recent research underscores the significance of complementary therapies and natural ingredients, such as French maritime pine bark extract (Malekahmadi et al. 2021), flavonoids (Maaliki et al. 2019), *Momordica charantia* L (Jandari et al. 2020), chia seed (Fateh et al. 2024; Karimi et al. 2024), and curcumin (Gowhari Shabgah et al. 2021; Vajdi et al. 2024) in the modulation of inflammation and oxidative stress associated with chronic diseases. Among these, curcumin has garnered particular scientific attention due to its pronounced antioxidant and anti‐inflammatory properties, as well as the comparatively greater volume of studies.

One of the most popular herbs in recent studies is turmeric, a member of the Zingiberaceae family, which has been used in Asian cultures for centuries (Prasath et al. 2024). Its distinctive orange‐yellow color is attributed to curcuminoids, lipophilic polyphenolic compounds that constitute approximately 2%–8% of turmeric's composition (Slaček et al. 2023). Curcuminoids display a diverse range of beneficial activities, encompassing antioxidant, anti‐inflammatory, anti‐tumor, antibacterial, cardio‐protective, and lipid‐lowering effects (Aminnezhad et al. 2023; Rapti et al. 2024; Kaur et al. 2024). The primary advantage of curcuminoids lies in their safety and tolerability in humans despite their limited bioavailability due to poor water solubility and rapid metabolism, which might decrease their suitability for many medical purposes (Zhao et al. 2024). However, it is confirmed that their bioavailability can be significantly enhanced by up to 2000% within 45 min in humans when administered orally in combination with piperine (Bertoncini‐Silva et al. 2024). Piperine, a bioactive alkaloid responsible for black pepper's pungency, has a diverse array of physiological effects, including antioxidant, anti‐obesity, cardioprotective, hepatoprotective, and anti‐inflammatory properties (Singh and Shukla 2024; Zare et al. 2025). Piperine can enhance the bioavailability of curcuminoids by increasing intestinal blood flow, enhancing enterocyte permeability, and reducing the activity of glucuronidase enzymes without inducing adverse effects (Abughazala 2024).

A systematic review involving 228 patients with non‐alcoholic fatty liver disease (NAFLD) demonstrated that supplementation with curcumin or turmeric significantly reduced ALT and AST levels when administered at high doses exceeding 1000 mg/day over 8 weeks. However, this analysis was based on only four studies and did not investigate the combined effects of Cur + Pip, which may explain the observed dose‐dependent efficacy of higher curcumin concentration (Mansour‐Ghanaei et al. 2019). Another study reported a significant decrease in IL‐6 concentrations after supplementing with Cur + Pip (Derosa et al. 2016).

In light of the recent surge in research within this field, it is imperative to undertake a meta‐analysis that integrates these novel studies, thereby enabling a more exhaustive and cohesive examination of the existing evidence. To the best of our knowledge, there is no comprehensive analysis evaluating the combined impact of Cur + Pip on liver enzymes and inflammatory markers. Hence, this meta‐analysis aims to systematically review the effectiveness of these co‐supplementations on liver enzymes and inflammatory markers in adults.

## 2 Methods

### 2.1 Study Design and Protocol

This systematic review and meta‐analysis was conducted in accordance with the principles outlined in the Preferred Reporting Items for Systematic Reviews and Meta‐Analyses (PRISMA) guidelines (Page et al. 2021). This study aims to meta‐analyze randomized controlled trials (RCTs) to evaluate the effectiveness of Cur + Pip on liver enzymes and inflammatory markers in adults.

The Participant, Intervention, Comparison/Control, Outcome (PICO) framework was employed in this study: the participants (P) are adults, and the intervention (I) under investigation is the effect of Cur + Pip. The comparison (C) group includes participants receiving either a placebo, a control treatment, or no treatment. The primary outcome (O) is the change in serum liver enzymes and inflammatory markers, including alanine transaminase (ALT), aspartate transaminase (AST), alkaline phosphatase (ALP), C‐reactive protein (CRP), Interleukin‐6 (IL‐6), and tumor necrosis factor‐alpha (TNF‐α) serum levels.

#### 2.1.1 Search Strategy

A comprehensive literature search was performed across three primary databases—MEDLINE/PubMed, ISI Web of Science, and Scopus—covering all available studies up to April 1, 2025. The search aimed to identify RCTs evaluating the effects of Cur + Pip on liver enzymes and inflammatory markers in adults. A combination of Medical Subject Headings (MeSH) terms and relevant keywords was used to find any studies investigating Cur + Pip, with AST, ALT, APP, CRP, IL‐6, and TNF‐α as primary outcomes. A complete list of keywords and search lines used in the search is provided in Tables S1–S3. To enhance the thoroughness of the review, Google Scholar and reference lists of the selected articles were manually screened for any additional relevant studies that may have been missed in the initial database search. No restrictions were applied regarding language or publication date, ensuring a broad and inclusive selection of studies.

### 2.2 Eligibility Criteria

The eligibility criteria for this study were established using the PICO framework to ensure a systematic selection of relevant studies. Adults were included as eligible participants, with the intervention of interest being Cur + Pip, compared to placebo, control groups, or no treatment. The primary outcomes assessed were changes in liver enzymes and inflammatory markers. Only RCTs were included, whereas non‐randomized studies, animal studies, in vitro studies, case series, case–control studies, cohort studies, abstracts, letters, systematic reviews, and meta‐analyses were excluded. No restrictions were applied regarding the time frame, language, or participant demographics such as sex, age, ethnicity, or comorbidities, allowing for a broad yet targeted analysis.

### 2.3 Data Extraction

Two reviewers (M.J. and F.V.) independently extracted data from the selected studies using a standardized Excel spreadsheet designed to ensure consistency and accuracy. The extracted information included essential study details such as the first author's name, year of publication, and study location, along with methodological aspects like study design, sample size, and characteristics of participants (mean age, sex, and BMI). Additionally, data on the Cur + Pip as an intervention were recorded, including dosage, trial duration, and observed changes in liver enzymes and inflammatory markers. Information about the control group was also documented for comparative analysis. Any variations or disagreements in the relevance or interpretation of the data were resolved through consultations with a third reviewer (M.K.) to guarantee accuracy and reach a consensus.

### 2.4 Quality Assessment

The quality assessment of the included randomized controlled trials (RCTs) was independently performed by two reviewers using the Cochrane risk of bias criteria, specifically employing version 2 of the Risk of Bias (RoB 2) tool (Flemyng et al. 2023). This assessment evaluated each study across five key domains: (D1) bias arising from the randomization process, (D2) deviations from intended interventions, (D3) missing outcome data, (D4) measurement of the outcome, and (D5) selection of the reported result. Each domain was rated as having a “Low risk,” “High risk,” or “Some concerns” regarding bias. Any disagreements between reviewers were resolved through discussion or consultation with a third reviewer to ensure consistency and objectivity in the quality appraisal.

### 2.5 Certainty Assessment

The GRADE (Appraisal, Development, and Evaluation of Recommendations) approach was used to evaluate and summarize the overall certainty of all included studies based on factors like study design, bias, and consistency. Evidence was categorized into four levels (high to very low) to ensure a transparent evaluation. This method helps synthesize research, guide clinical decisions, and support evidence‐based guidelines (Guyatt et al. 2008).

### 2.6 Statistical Analyses

Statistical analyses were conducted using Stata version 11.1 (Stata Corp, College Station, TX), with a significance threshold of *p* < 0.05 for all two‐tailed tests. A random‐effects model was employed to calculate pooled weighted mean differences (WMD), accounting for heterogeneity (DerSimonian and Laird 1986). Mean differences (MD) in outcomes were determined between baseline and post‐intervention for the intervention and control groups. The standard deviation (SD) of mean differences was derived using the formula: SD = √[(SD at baseline)^2^ + (SD at the end)^2^ − (2 × *r* × SD at baseline × SD at the end)] (Asbaghi et al. 2020), with a correlation coefficient (*r*) of 0.8. For studies reporting standard errors (SE) instead of SD, the Hozo et al. (2005) method was used to convert SE, 95% confidence intervals (CI), and interquartile ranges into SDs. Heterogeneity was evaluated using Cochrane's *Q* test and the *I* ^2^ statistic, with significant heterogeneity defined as *I* ^2^ > 40%. Publication bias was assessed via visual inspection of funnel plots and Egger's and Begg's tests, with sensitivity analyses performed using the leave‐one‐out method (Begg and Mazumdar 1994; Egger et al. 1997). Meta‐regression was employed to assess the effects of intervention dose and duration on outcomes. The trim‐and‐fill method was applied to address publication bias (Duval 2005).

## 3 Results

### 3.1 Study Selection

**FIGURE 1:**
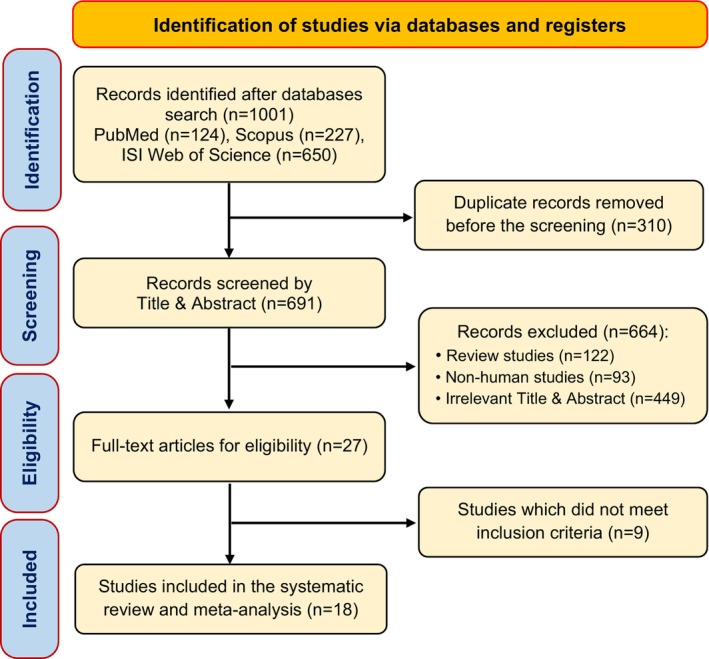
PRISMA Flow chart of study selection for inclusion trials in the systematic review.

The study selection process commenced with the identification of 1001 records through database searches, including PubMed (*n* = 124), ISI Web of Science (*n* = 227), and Scopus (*n* = 650). After eliminating 310 duplicate entries, 691 documents underwent screening. Based on title and abstract relevance, 664 articles were excluded for not aligning with the research topic. This left 27 full‐text articles for further eligibility assessment. Ultimately, nine studies were excluded due to insufficient or missing relevant data, lack of a control group, or no combination of both supplements, leading to the inclusion of 18 RCTs in the systematic review (Panahi et al. 2018, 2019, 2015; Saberi‐Karimian et al. 2020; Cicero et al. 2020; Mirhafez et al. 2021; Tabaee et al. 2021; Askari et al. 2022; Arabnezhad et al. 2022; Sharifi et al. 2023; Mohammadi et al. 2013; Rahimnia et al. 2015; Zahedi et al. 2021; Boshagh et al. 2023; Amini et al. 2024; Hosseini et al. 2024; Ganjali et al. 2014; Miranda‐Castro et al. 2022). The study flowchart is depicted in Figure 1.

### 3.2 Study Characteristics

Table 1 summarizes the basic characteristics of the included studies (Panahi et al. 2018, 2019, 2015; Saberi‐Karimian et al. 2020; Cicero et al. 2020; Mirhafez et al. 2021; Tabaee et al. 2021; Askari et al. 2022; Arabnezhad et al. 2022; Sharifi et al. 2023; Mohammadi et al. 2013; Rahimnia et al. 2015; Zahedi et al. 2021; Boshagh et al. 2023; Amini et al. 2024; Hosseini et al. 2024; Ganjali et al. 2014; Miranda‐Castro et al. 2022). A total of 18 RCTs with 18 effect sizes assessed liver enzymes and inflammatory markers. Specifically, nine effect sizes were reported for AST and ALT (Panahi et al. 2018, 2019; Saberi‐Karimian et al. 2020; Cicero et al. 2020; Mirhafez et al. 2021; Tabaee et al. 2021; Askari et al. 2022; Arabnezhad et al. 2022; Sharifi et al. 2023), 4 for ALP (Panahi et al. 2019; Mirhafez et al. 2021; Tabaee et al. 2021; Arabnezhad et al. 2022), 10 for CRP (Panahi et al. 2018, 2015; Askari et al. 2022; Sharifi et al. 2023; Mohammadi et al. 2013; Rahimnia et al. 2015; Zahedi et al. 2021; Boshagh et al. 2023; Amini et al. 2024; Hosseini et al. 2024), 4 for IL‐6 (Rahimnia et al. 2015; Zahedi et al. 2021; Ganjali et al. 2014; Miranda‐Castro et al. 2022), and 5 for TNF‐α (Saberi‐Karimian et al. 2020; Rahimnia et al. 2015; Zahedi et al. 2021; Ganjali et al. 2014; Miranda‐Castro et al. 2022). The studies were conducted between 2012 and 2024 and involved 1063 participants, 531 in the intervention group and 532 in the placebo group.

**TABLE 1:** Basic characteristics of the included studies in the meta‐analysis.

Study	Country	Study design	Participant	Gender (m/f)	Sample size (int./cont.)	Trial duration	Age (int.)	Age (cont.)	BMI (int.)	BMI (cont.)	Intervention (g/day)	Control
Mohammadi et al. (2013)	Iran	Cross‐over, R, PC, DB	Obese patients	M: 0 F: 30	30 (15/15)	4 weeks	38/43 ± 10/84	38.43 ± 10.84	32/56 ± 3/58	32/56 ± 3/58	Curcumin + piperine (1000 + 10 mg/day)	Placebo
Ganjali et al. (2014)	Iran	Cross‐over, R, PC, DB	Obese individuals	NR	30 (15/15)	4 weeks	18–65	18–65	≥ 30	≥ 30	Curcumin + piperine (1000 + NR mg/day)	Placebo
Panahi et al. (2015)	Iran	Parallel, R, PC, DB	Metabolic syndrome	M: 20 F: 20	78 (39/39)	8 weeks	33.55 ± 9.44	38.75 ± 8.04	25.46 ± 2.46	22.8 ± 5.37	Curcumin + piperine (1000 + 10 mg/day)	Placebo
Rahimnia et al. (2015)	Iran	Parallel, R, PC, DB	Knee osteoarthritis	M: 9 F: 31	40 (19/21)	6 weeks	36.51 ± 9.44	35.43 ± 10.04	28.75 ± 3.17	29.64 ± 4.46	Curcumin + piperine (1500 + 15 mg/day)	Placebo
Panahi et al. (2018)	Iran	Parallel, R, PC, DB	T2DM	M: 51 F: 49	100 (50/50)	12 weeks	43 ± 8	41 ± 7	26 ± 2	27 ± 2	Curcumin + piperine (500 + 5 mg/day)	Placebo
Panahi et al. (2019)	Iran	Parallel, R, PC	NAFLD	M: 39 F: 31	70 (35/35)	12 weeks	46.63 ± 13.7	47.51 ± 14.49	NR	NR	Curcumin + piperine (500 + 5 mg/day)	Placebo
Saberi‐Karimian et al. (2020)	Iran	Parallel, R, PC, DB	NAFLD	M: NR F: NR	49 (23/26)	8 weeks	18–70	18–70	30.16 ± 4.14	30.02 ± 5.45	Curcumin + piperine (500 + 5 mg/day)	Placebo
Cicero et al. (2020)	Italy	Parallel, R, PC, DB	Suboptimal fasting plasma glucose	M: 37 F: 43	80 (40/40)	8 weeks	54 ± 3	53 ± 5	27.1 ± 1.8	26.9 ± 1.9	Curcumin + piperine (800 + 8 mg/day)	Placebo
Mirhafez et al. (2021)	Iran	Parallel, R, PC, DB	NAFLD	M: 40 F: 30	70 (37/33)	8 weeks	45.6 ± 11.0	43.1 ± 11.6	30.9 ± 4.3	29.2 ± 4.2	Curcumin + piperine (500 + 5 mg/day)	Placebo
Tabaee et al. (2021)	Iran	Parallel, R, PC, DB	Acute myocardial infarction	M: 45 F: 27	72 (38/34)	8 weeks	59.5 ± 10.4	59.6 ± 10.3	26.0 ± 4.4	26.0 ± 4.9	Curcumin + piperine (500 + 5 mg/day)	Placebo
Zahedi et al. (2021)	Iran	Parallel, R, PC, DB	Critically ill patients with tuberculosis infection	M: 49 F: 13	62 (31/31)	1 week	40.8 ± 15.3	45.4 ± 15.8	25.4 ± 3.5	25.3 ± 3.3	Curcumin + piperine (500 + 5 mg/day)	Placebo
Askari et al. (2021)	Iran	Parallel, R, PC, DB	COVID‐19	M: 27 F: 19	46 (23/23)	2 weeks	43.74 ± 12.9	51.52 ± 13.8	26.74 ± 3.9	26.35 ± 4.7	Curcumin + piperine (500 + 5 mg/day)	Placebo
Miranda‐Castro et al. (2022)	Brazil	Cross‐over, R, PC, DB	Healthy, runners	M: 16 F: 0	16 (8/8)	1 week	36 ± 9	36 ± 9	NR	NR	Curcumin + piperine (500 + 20 mg/day)	Placebo
Arabnezhad et al. (2022)	Iran	Parallel, R, PC, TB	Premenstrual syndrome and dysmenorrhea	M: 0 F: 76	76 (38/38)	12 weeks	18–24	18–24	21.3 ± 2.3	20.8 ± 3.9	Curcumin + piperine (500 + 5 mg/day)	Placebo
Boshagh et al. (2023)	Iran	Parallel, R, PC, DB	Ischemic stroke in the rehabilitation phase	M: 26 F: 40	56 (27/29)	12 weeks	59.48 ± 5.15	60.12 ± 3.12	26.61 ± 4.84	27.04 ± 3.79	Curcumin + piperine (500 + 5 mg/day)	Placebo
Amini et al. (2024)	Iran	Parallel, R, PC, DB	Non‐proliferative diabetic retinopathy	M: 19 F: 37	56 (27/29)	12 weeks	55.85 ± 8.22	55.86 ± 7.99	27.87 ± 5.02	27.93 ± 3.76	Curcumin + piperine (1000 + 10 mg/day)	Placebo
Sharifi et al. (2023)	Iran	Parallel, R, PC, DB	Hepatic steatosis	M: 36 F: 24	60 (30/30)	12 weeks	43 ± 9.72	47.5 ± 13.31	29.46 ± 3.34	29.36 ± 4.29	Curcumin + piperine (500 + 5 mg/day)	Placebo
Hosseini et al. (2024)	Iran	Parallel, R, PC, DB	T2DM + hypertriglyceridemia	M: 22 F: 50	72 (36/36)	12 weeks	55.25 ± 7.46	77.30 ± 13.40	31.55 ± 6.42	30.55 ± 6.02	Curcumin + piperine (500 + 5 mg/day)	Placebo

Abbreviations: Cont., control; DB, double‐blind; F, female; Int., intervention; M, male; NAFLD, non‐alcoholic fatty liver disease; NR, not reported; PC, placebo‐controlled; R, randomized clinical trial; T2DM, type 2 diabetes mellitus.

Geographically, a significant proportion of the studies were conducted in Iran (Panahi et al. 2018, 2019, 2015; Saberi‐Karimian et al. 2020; Mirhafez et al. 2021; Tabaee et al. 2021; Askari et al. 2022; Arabnezhad et al. 2022; Sharifi et al. 2023; Mohammadi et al. 2013; Rahimnia et al. 2015; Zahedi et al. 2021; Boshagh et al. 2023; Amini et al. 2024; Hosseini et al. 2024; Ganjali et al. 2014), whereas others took place in Italy (Cicero et al. 2020) and Brazil (Miranda‐Castro et al. 2022). The majority of the included RCTs employed a parallel, double‐blind design; however, three studies conducted a crossover design (Mohammadi et al. 2013; Ganjali et al. 2014; Miranda‐Castro et al. 2022), and one study utilized a triple‐blind design (Arabnezhad et al. 2022). Participants' mean ages ranged from 18 to 77 years, and baseline BMI values varied between 22.8 and 32.56 kg/m^2^. The duration of supplementation across studies ranged from 1 to 12 weeks.

Most studies included both male and female participants, except for two studies that exclusively involved females (Arabnezhad et al. 2022; Mohammadi et al. 2013) and one study that included only male participants (Miranda‐Castro et al. 2022). All studies investigated the effects of daily supplementation with Cur + Pip as the intervention, compared with a placebo in the control group. The study populations encompassed individuals with diabetes (Panahi et al. 2018; Cicero et al. 2020; Amini et al. 2024; Hosseini et al. 2024), liver disease (Panahi et al. 2019; Saberi‐Karimian et al. 2020; Mirhafez et al. 2021; Sharifi et al. 2023), obesity (Mohammadi et al. 2013; Ganjali et al. 2014), central nervous system injuries (Zahedi et al. 2021; Boshagh et al. 2023), myocardial infarction (Tabaee et al. 2021), metabolic syndrome (Panahi et al. 2015), menstrual disorders (Arabnezhad et al. 2022), COVID‐19 (Askari et al. 2022), as well as athletes (runners) (Miranda‐Castro et al. 2022) and patients with knee osteoarthritis (Rahimnia et al. 2015).

### 3.3 Meta‐Analysis on Liver Enzymes

**FIGURE 2:**
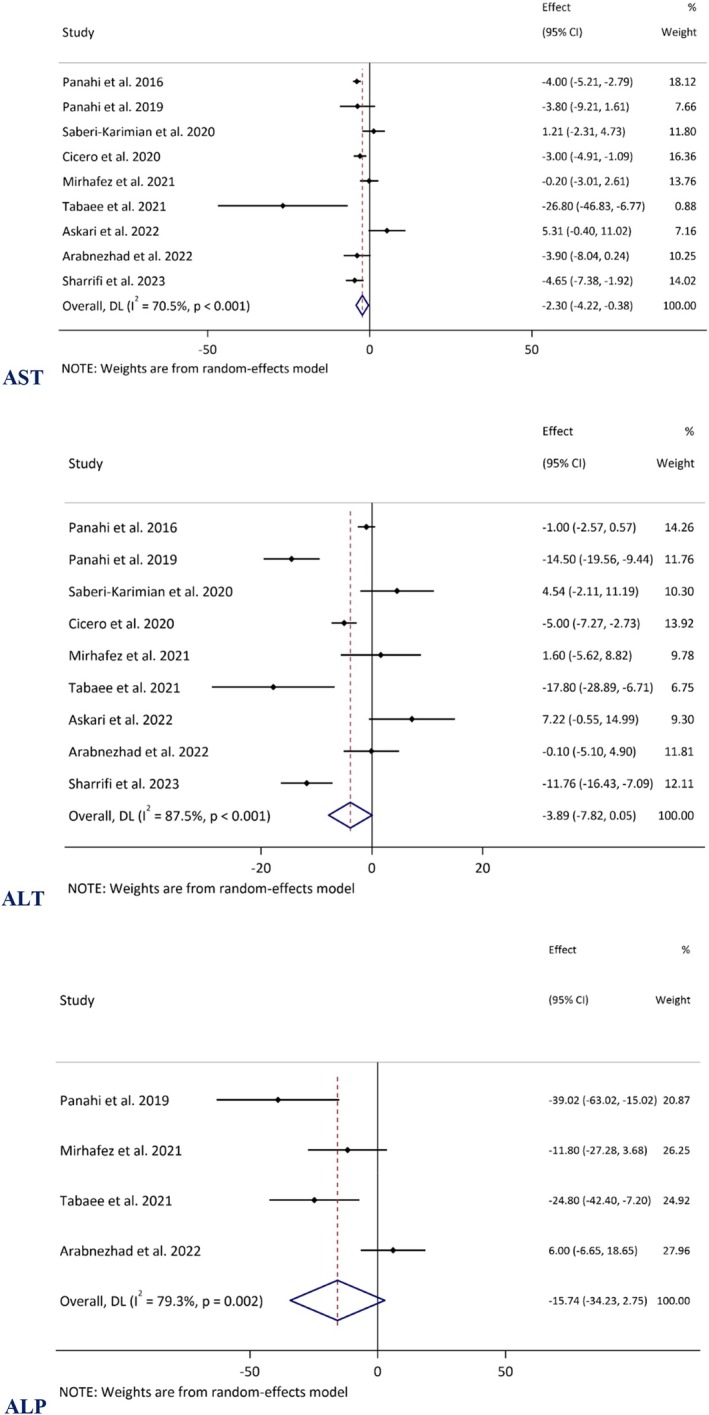
Forest plot demonstrating mean difference (MD) and 95% confidence interval (CIs) for the effect of curcuminoids plus piperine co‐supplementation on liver enzymes, including aspartate aminotransferase (AST), alanine aminotransferase (ALT), and alkaline phosphatase (ALP).

A meta‐analysis of data from nine RCTs (Panahi et al. 2018, 2019; Saberi‐Karimian et al. 2020; Cicero et al. 2020; Mirhafez et al. 2021; Tabaee et al. 2021; Askari et al. 2022; Arabnezhad et al. 2022; Sharifi et al. 2023) revealed that Cur + Pip significantly reduced serum AST levels (Panahi et al. 2018, 2019; Saberi‐Karimian et al. 2020; Cicero et al. 2020; Mirhafez et al. 2021; Tabaee et al. 2021; Askari et al. 2022; Arabnezhad et al. 2022; Sharifi et al. 2023). The WMD was −2.29 IU (95% CI: −4.21 to −0.37, *p* = 0.019), with substantial heterogeneity observed (*I* ^2^ = 70.5%, *p* = 0.001). Regarding ALT, the overall WMD was −3.88 IU (95% CI: −7.81 to 0.04, *p* = 0.053), showing substantial heterogeneity (*I* ^2^ = 87.5%, *p* < 0.001). Furthermore, an analysis of four RCTs assessing ALP levels demonstrated a WMD of −15.74 (95% CI: −34.23 to 2.74, *p* = 0.095) (Panahi et al. 2019; Mirhafez et al. 2021; Tabaee et al. 2021; Arabnezhad et al. 2022) (Figure 2).

Subgroup analyses of AST and ALT levels indicated a significant reduction in serum levels among individuals with metabolic dysfunction compared to other groups. Notably, overweight participants (BMI: 25–29.9 kg/m^2^) revealed favorable responses to co‐supplementation. Regarding trial duration, studies lasting 12 weeks or more demonstrated a statistically significant reduction in AST levels (WMD = −4.08 IU, 95% CI: −5.12 to −3.03, *p* < 0.001). However, the effect of prolonged Cur + Pip intake on ALT levels was only marginally significant (WMD = −6.68 IU, 95% CI: −13.71 to 0.35, *p* = 0.063). No significant associations were observed for AST and ALT levels in participants with a normal BMI or obesity, in shorter‐duration trials, or among comorbidities other than metabolic dysfunction (Table 2).

**TABLE 2:** Meta‐analysis findings of curcumin plus piperine co‐supplementations on liver enzymes and inflammatory markers in adults.

	No. of effect sizes	WMD (95% CI)	*p*	Heterogeneity		
				*p*‐heterogeneity	*I* ^2^	*p* between sub‐groups
Subgroup analyses of curcumin + piperine on serum ALT (IU)						
Overall effect	9	−3.88 (−7.81, 0.04)	0.053	< 0.001*	87.5%	
Trial duration (week)						
< 12	5	−1.30 (−7.94, 5.33)	0.700	< 0.001*	82.5%	0.276
≥ 12	4	−6.68 (−13.71, 0.35)	0.063	< 0.001*	92.7%	
Health						
Metabolic dysfunction	6	−4.64 (−9.15, −0.13)	0.044*	< 0.001*	89.8%	0.763
Other	3	−2.76 (−14.11, 8.59)	0.634	0.001*	84.8%	
Baseline BMI (kg/m^2^)						
Normal (18.9–24.9)	1	−0.10 (−5.10, 4.90)	0.969	—	—	0.069
Overweight (25–29.9)	5	−4.89 (−9.76, −0.03)	0.048*	< 0.001*	88.9%	
Obese (≥ 30)	2	3.19 (−1.70, 8.08)	0.201	0.557	0.0%	
Subgroup analyses of curcumin + piperine on AST (IU)						
Overall effect	9	−2.29 (−4.21, −0.37)	0.019*	0.001*	70.5%	
Trial duration (week)						
< 12	5	−0.47 (−4.04, 3.08)	0.794	0.002	76.4%	0.057
≥ 12	4	−4.08 (−5.12, −3.03)	< 0.001*	0.977	0.0%	
Health						
Metabolic dysfunction	6	−2.60 (−4.26, −0.93)	0.002*	0.021	62.3%	0.774
Other	3	−4.17 (−14.78, 6.43)	0.441	0.002	84.5%	
Baseline BMI (kg/m^2^)						
Normal (18.9–24.9)	1	−3.90 (−8.03, 0.23)	0.065	—	—	0.066
Overweight (25–29.9)	5	−3.04 (−5.60, −0.49)	0.020*	0.003	74.8%	
Obese (≥ 30)	2	0.35 (−1.84, 2.54)	0.755	0.539	0.0%	
Subgroup analyses of curcumin + piperine on serum ALP (IU)						
Overall effect	4	−15.74 (−34.23, 2.74)	0.095	0.002	79.3%	
Subgroup analyses of curcumin + piperine on serum CRP (mg/dL)						
Overall effect	10	−1.53 (−3.99, 0.92)	0.222	< 0.001*	98.5%	
Baseline (mg/dL)						
< 3	2	−0.77 (−2.80, 1.24)	0.450	0.123	57.9%	0.621
> 3	8	−1.66 (−4.53, 1.20)	0.256	< 0.001*	98.7%	
Trial duration (week)						
< 12	5	−2.14 (−5.86, 1.57)	0.258	< 0.001*	99.1%	0.308
≥ 12	5	−0.19 (−0.72, 0.34)	0.485	0.453	0.0%	
Dose (mg/day)						
Curcumin + piperine (500 + 5)	6	−2.44 (−6.85, 1.95)	0.276	< 0.001*	98.7%	0.686
Curcumin + piperine (1000 + 10)	3	−0.46 (−2.51, 1.57)	0.653	< 0.001*	94.6%	
Curcumin + piperine (1500 + 15)	1	−0.45 (−1.48, 0.58)	0.396	—	—	
Health						
Metabolic dysfunction	7	−0.58 (−1.74, 0.57)	0.321	< 0.001*	87.6%	0.456
Other	3	−3.03 (−9.36, 3.29)	0.348	< 0.001*	98.9%	
Baseline BMI (kg/m^2^)						
Overweight (25–29.9)	7	−1.63 (−4.51, 1.24)	0.267	< 0.001*	98.9%	0.145
Obese (≥ 30)	2	0.62 (−0.32, 1.56)	0.197	0.374	0.0%	
Subgroup analyses of curcumin + piperine on serum IL‐6 (pg/mL)						
Overall effect	4	−5.59 (−8.57, −2.61)	< 0.001*	< 0.001*	96.9%	
Subgroup analyses of curcumin + piperine on serum TNF‐α (pg/mL)						
Overall effect	5	−0.77 (−2.29, 0.73)	0.314	< 0.001	91.1%	

Abbreviations: ALP, alkaline phosphatase; ALT, alanine transaminase; AST, aspartate aminotransferase; BMI, body mass index; CI, confidence interval; CRP, C‐reactive protein; IL‐6, interleukin‐6; TNF‐α, tumor necrosis factor‐alpha; WMD, weighted mean differences. * and Bold values are statistically significant (*p* < 0.05).

### 3.4 Effects on Inflammatory Markers (CRP, IL‐6, TNF‐α)

**FIGURE 3:**
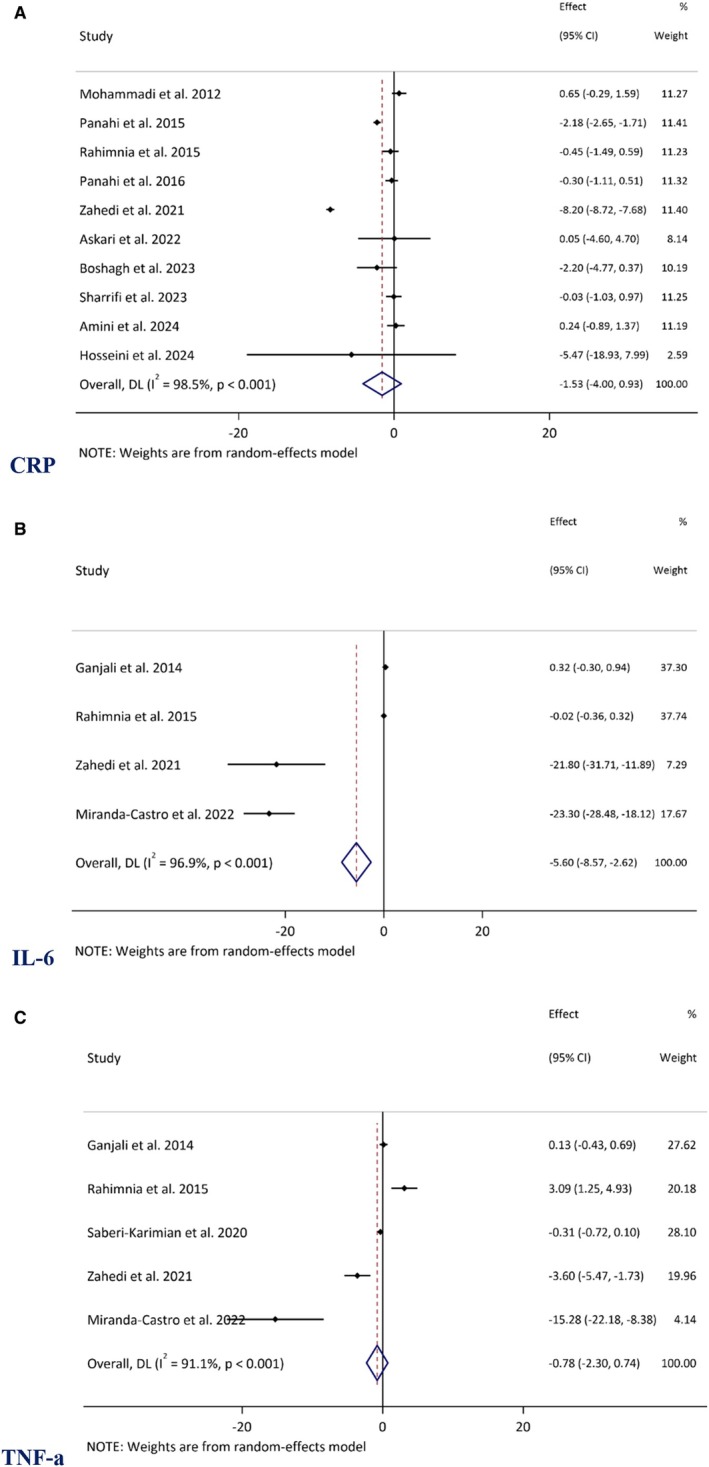
Forest plot demonstrating mean difference (MD) and 95% confidence interval (CIs) for the effect of curcuminoids plus piperine co‐supplementation on (A) C‐reactive protein (CRP), (B) interleukin‐6 (IL‐6), (C), and tumor necrosis factor‐alpha (TNF‐α).

A total of ten RCTs were included in the meta‐analysis of CRP levels (Panahi et al. 2018, 2015; Askari et al. 2022; Sharifi et al. 2023; Mohammadi et al. 2013; Rahimnia et al. 2015; Zahedi et al. 2021; Boshagh et al. 2023; Amini et al. 2024; Hosseini et al. 2024). As presented in Figure 3, the overall WMD was −1.53 mg/dL (95% CI: −3.99 to 0.92, *p* = 0.222), indicating a non‐significant decrease in CRP levels. The analysis demonstrated considerable heterogeneity (*I* ^2^ = 98.5%, *p* < 0.001).

In contrast, a statistically significant reduction in IL‐6 levels was observed following supplementation. A pooled analysis of four RCTs reported a WMD of −5.59 pg/mL (95% CI: −8.57 to −2.61, *p* < 0.001) (Rahimnia et al. 2015; Zahedi et al. 2021; Ganjali et al. 2014; Miranda‐Castro et al. 2022). However, based on data from five RCTs, no significant association was found between Cur + Pip and changes in TNF‐α levels (WMD: −0.77 pg/mL, 95% CI: −2.29 to 0.73, *p* = 0.314) (Saberi‐Karimian et al. 2020; Rahimnia et al. 2015; Zahedi et al. 2021; Ganjali et al. 2014; Miranda‐Castro et al. 2022). This analysis also exhibited considerable heterogeneity (*I* ^2^ = 91.1%, *p* < 0.001) (Figure 3).

The results of subgroup analyses based on baseline serum CRP levels, trial duration, supplementation dosage, health status, and baseline BMI revealed no statistically significant modifying effect (Table 2).

### 3.5 Publication Bias

**FIGURE 4:**
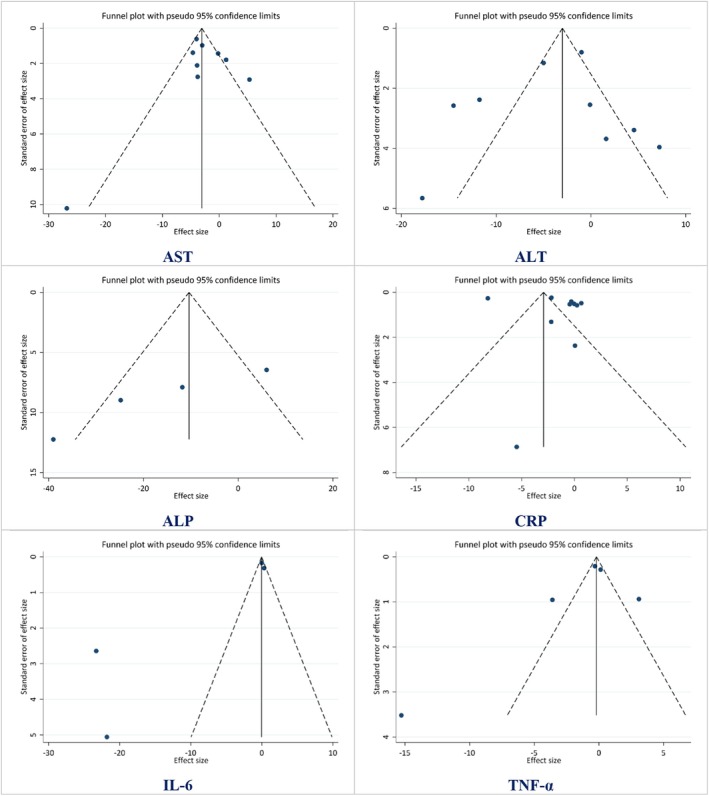
Funnel plot demonstrating publication bias for the effect of curcuminoids plus piperine co‐supplementation on liver enzymes, including aspartate aminotransferase (AST), alanine aminotransferase (ALT), alkaline phosphatase (ALP), C‐reactive protein (CRP), Interleukin‐6 (IL‐6), and tumor necrosis factor‐alpha (TNF‐α) in adults.

Publication bias was assessed for each outcome, with *p* values indicating the likelihood of bias in the included studies. Most outcomes, including CRP (*p* = 0.324), IL‐6 (*p* = 0.132), TNF‐α (*p* = 0.498), ALT (*p* = 0.601), and AST (*p* = 0.606), showed no significant evidence of publication bias. However, ALP demonstrated potential publication bias with a statistically significant *p* value (*p* = 0.041) (Figure 4) (Table S3).

### 3.6 Sensitivity Analysis

A sensitivity analysis assessed the impact of individual studies on overall results. No analysis was conducted for CRP due to minimal variation. For IL‐6, excluding studies like Ganjali et al. (2014) and Rahimnia et al. (2015) resulted in effect size changes (95% CI: −33.43 to 3.79). TNF‐α showed minor shifts (95% CI: −3.12 to −0.06) with Rahimnia et al. (2015). ALT results remained stable across multiple studies (95% CI: −9.03 to −0.04). AST was influenced by several studies, with variations (95% CI: −4.55 to 0.47). ALP showed the most sensitivity, particularly with Arabnezhad et al. (2022) (WMD: −23.08, 95% CI: −37.69, −8.47), indicating its strong influence on the outcome. Overall, most results remained stable, with some studies contributing to heterogeneity (Table S3).

### 3.7 Risk of Bias Assessment

**FIGURE 5:**
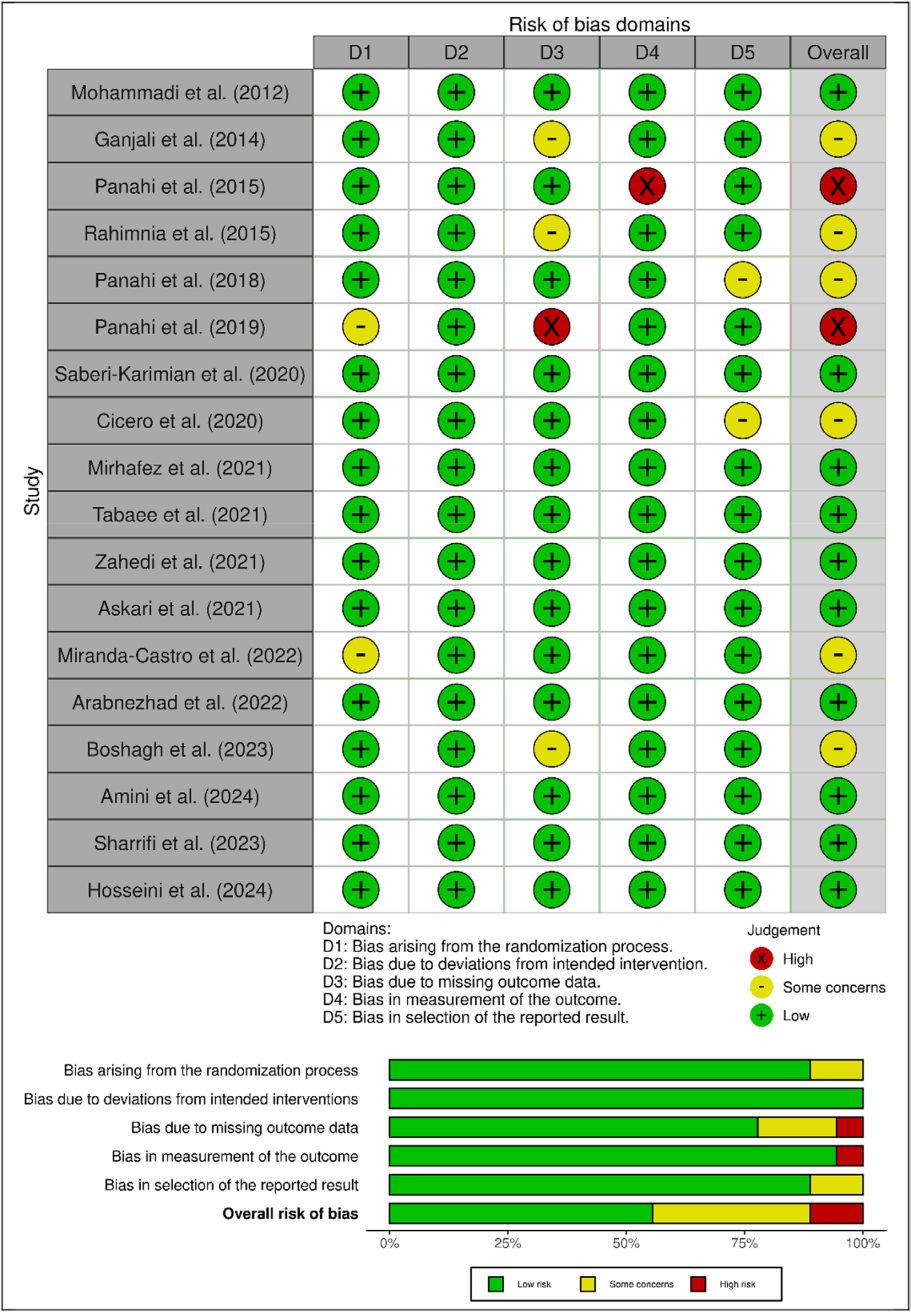
Risk of bias (RoB) assessment of studies in the meta‐analysis.

The risk of bias assessment for the included studies demonstrates that the majority had a low risk of bias across all domains. Most studies exhibited green (+) ratings, indicating a low risk in key areas such as the randomization process (D1), deviations from the intended intervention (D2), missing outcome data (D3), measurement of outcomes (D4), and selection of reported results (D5). However, a few studies, such as Panahi et al. (2019) and Panahi et al. (2015), showed a high risk of bias (red) in D4 and overall assessment, mainly due to concerns in outcome measurement. Additionally, some studies, including Panahi et al. (2019), Miranda‐Castro et al. (2022), and Boshagh et al. (2023), had moderate concerns (yellow) in D1 and D3. Overall, whereas most studies were judged to have a low risk of bias, a few exhibited moderate to high risks, particularly related to the measurement of outcomes and missing data (Figure 5).

### 3.8 GRAID Assessment

Table 3 represents the GRADE assessment of Cur + Pip on liver enzymes and inflammatory markers, indicating varying levels of evidence quality. While the risk of bias and indirectness was generally not serious across all outcomes, inconsistency and imprecision were notable concerns. ALT, ALP, CRP, IL‐6, and TNF‐α showed very serious inconsistency, contributing to lower evidence quality, with ALT, ALP, CRP, and TNF‐α also exhibiting serious imprecision. AST had serious inconsistency but no issues with imprecision, maintaining a high‐quality rating (⊕⊕⊕⊕). In contrast, ALP had the lowest evidence rating (⊕⊕⊖⊖, very low) due to very serious inconsistency, serious imprecision, and a serious limitation in publication bias. IL‐6 received a moderate rating (⊕⊕⊕⊖), whereas ALT, CRP, and TNF‐α were rated low (⊕⊕⊖⊖), reflecting the impact of serious limitations. These findings suggest that while AST results are more reliable, other markers have lower confidence due to inconsistency and imprecision in the data.

**TABLE 3:** GRADE assessment of curcumin plus piperine co‐supplementations on liver enzymes and inflammatory markers.

Outcomes	Risk of bias	Inconsistency	Indirectness	Imprecision	Publication bias	Quality of evidence
ALT	No serious limitation	Very serious limitation^1^	No serious limitation	Serious limitation^3^	No serious limitation	⊕◯◯◯ Low
AST	No serious limitation	Serious limitation^2^	No serious limitation	No serious limitation	No serious limitation	⊕⊕⊕⊕◯ High
ALP	No serious limitation	Very serious limitation^1^	No serious limitation	Serious limitation^3^	Serious limitation^4^	◯◯◯◯ Very low
CRP	No serious limitation	Very serious limitation^1^	No serious limitation	Serious limitation^3^	No serious limitation	⊕◯◯◯ Low
IL‐6	No serious limitation	Very serious limitation^1^	No serious limitation	No serious limitation	No serious limitation	⊕⊕◯◯ Moderate
TNF‐α	No serious limitation	Very serious limitation^1^	No serious limitation	Serious limitation^3^	No serious limitation	⊕◯◯◯ Low

Abbreviations: ALP, alkaline phosphatase; ALT, alanine transaminase; AST, aspartate aminotransferase; CRP, C‐reactive protein; IL‐6, interleukin‐6; TNF‐α, tumor necrosis factor‐alpha. ^1^There is a high heterogeneity (I2 > 75%); ^2^There is a moderate heterogeneity (I2 > 40%);^3^There is no evidence of significant effects of Curcumin Plus Piperine; ^4^There is a significant publication bias based on Egger’s test.

## 4 Discussion

### 4.1 Aim and Main Findings

This systematic review aimed to meta‐analyze RCTs to evaluate the effectiveness of Cur + Pip on liver enzymes and inflammatory markers in adults. The findings indicate that this co‐supplementation improved the liver enzyme AST. However, the effect on ALT and ALP was not statistically significant, with a *p* value of 0.53 for ALT, suggesting a trend that approached significance. Regarding inflammatory markers, the Cur + Pip led to a statistically significant decrease in IL‐6 concentrations, whereas no significant effects were observed on CRP and TNF‐α levels. Subgroup analysis revealed a potential benefit of this co‐supplementation for overweight and/or metabolic dysfunction individuals, as evidenced by significant reductions in ALT and AST levels. Furthermore, a supplementation duration exceeding 12 weeks was associated with improved AST levels. Although the reduction in ALT levels following longer‐term supplementation was not statistically significant, it exhibited a more substantial decrease in serum levels compared to AST (−6.68 IU vs. −4.08 IU, respectively).

Chronic inflammation, along with degeneration, scarring, and fibrosis of hepatic cells, constitutes a gradual process that ultimately leads to hepatic steatosis over an extended period (Varra et al. 2024). As fibrosis progresses, it can result in cirrhosis, which is characterized by irreversible damage to liver cells (Wang et al. 2024). Consequently, identifying an effective treatment to halt this progression is crucial.

Nevertheless, recent research has highlighted the growing significance of herbal products in enhancing health outcomes, reflecting increased integration of phytotherapy into modern healthcare (Sharifi et al. 2024). This trend is driven by concerns over conventional drug side effects, a focus on preventive care, and accumulating evidence supporting the efficacy and safety of herbal medicines (Hashempur et al. 2019; Meysami et al. 2021). Therefore, herbal therapies are increasingly recognized as valuable components of contemporary clinical practice.

Previous meta‐analyses have examined the impact of curcumin supplementation, either alone or in combination with piperine, on liver enzyme levels in patients with NAFLD. Evidence suggests that curcumin supplementation exerts beneficial effects on AST and ALT levels, whereas no significant effect has been observed on ALP levels (Ebrahimzadeh et al. 2025; Aragón‐Vela et al. 2024). Additionally, one study reported that the Cur + Pip did not significantly influence aminotransferase levels in NAFLD patients (Vajdi et al. 2024). Considering inflammation, one study demonstrated that the supplementation of curcumin in conjunction with piperine significantly reduced concentrations of TNF‐α and IL‐6 in both healthy and unhealthy populations (Hosseini et al. 2025). Conversely, a separate analysis encompassing 19 studies found no significant impact of turmeric or curcumin supplementation on various inflammatory markers, including CRP, hs‐CRP, IL‐1β, IL‐6, and TNF‐α, among patients with chronic inflammatory conditions (White et al. 2019). The inconsistency observed in these findings could be attributed to potential sources of heterogeneity among the studies, including differences in the duration of the interventions, the dosage of curcumin administered, or whether curcumin was used alone or in combination with piperine. Moreover, the present study offers a more comprehensive analysis by incorporating a larger number of RCTs and including all adult populations. However, prior meta‐analyses have been limited to NAFLD patients and chronic inflammatory conditions.

Curcumin supplementation exerts its effects on liver enzymes and inflammation through multiple mechanisms, but its clinical efficacy is often limited by its poor oral bioavailability, which is less than 2% due to low absorption, rapid metabolism, and systemic elimination (Tabanelli et al. 2021). On the other hand, piperine, a bioactive alkaloid derived from black pepper, significantly enhances curcumin's bioavailability by inhibiting key hepatic and intestinal enzymes involved in its metabolism, including UDP‐glucuronosyltransferase (UGT), cytochrome P450 enzymes (notably CYP3A4), and P‐glycoprotein (Gorgani et al. 2017). This enzymatic inhibition reduces curcumin's metabolic clearance, resulting in increased plasma concentrations, reported to be up to 20‐fold higher in humans, thereby amplifying its biological effects (Racz et al. 2022). Beyond bioavailability enhancement, piperine exerts antioxidant effects by activating intracellular pathways such as nuclear factor erythroid 2‐related factor 2 (Nrf2) and peroxisome proliferator‐activated receptor gamma (PPAR‐γ), which promote cellular antioxidant defenses and reduce oxidative stress (Mirhafez et al. 2019). It also modulates inflammatory signaling by inhibiting nuclear factor‐kappa B (NF‐κB), NLRP3 inflammasome, and mitogen‐activated protein kinases (MAPKs), thereby attenuating pro‐inflammatory cytokine production (Dludla et al. 2023).

Curcumin could act as a potent antioxidant, effectively reducing oxidative stress in liver cells (Kaur et al. 2024). By scavenging free radicals and decreasing lipid peroxidation, curcumin protects hepatocytes from injury (Yang et al. 2024). Curcumin also inhibits the nuclear factor kappa B (NF‐κB) signaling pathway, a key regulator of inflammation, which results in the reduction of the expression of pro‐inflammatory cytokines and improvement in liver enzymes (Ayubi et al. 2024). Moreover, it inhibits the activation of hepatic stellate cells (HSCs), which are responsible for extracellular matrix deposition during liver injury (Xie et al. 2024). It interferes with several signaling pathways involved in HSC activation, including those mediated by leptin and transforming growth factor‐beta (TGF‐β), thereby preventing fibrosis progression (Abolfazli et al. 2025). Curcumin influences lipid metabolism by reducing triglyceride synthesis through the inhibition of enzymes like HMG‐CoA reductase and enhancing the expression of CYP7A1, an enzyme involved in cholesterol metabolism. This regulation aids in clearing excess fat from liver cells (Unhapipatpong et al. 2025). Despite its numerous benefits, curcumin does not significantly alter ALP and ALT levels. This may be due to the inherent properties of curcumin and its metabolic pathways, which may not directly influence the level of ALP and ALT (Guariglia et al. 2023). Overall, curcumin could be a promising therapeutic intervention for various liver diseases characterized by inflammation and fat accumulation (Qin et al. 2024).

Inflammation represents a complex biological response mediated by the immune system, designed to prevent, limit, and repair damage caused by invading pathogens or endogenous biomolecules (Gusev and Zhuravleva 2022). While acute inflammation is a transient and beneficial response, chronic inflammation is associated with tissue dysfunction and pathology, characterized by an imbalance between pro‐ and anti‐inflammatory processes, abnormal cytokine levels, and elevated production of acute‐phase reactants such as CRP (Leyane et al. 2022). Key cytokines involved include TNF‐α, IL‐6, and IL‐1β, which are pivotal in the interplay between metabolic disease, inflammation, and carcinogenesis (Zhao et al. 2021). TNF‐α plays a crucial role in modulating inflammation across various diseases, with its activity regulated by the NF‐κB transcription factor (Belenguer et al. 2021). Furthermore, inflammatory processes are closely linked to oxidative stress, where the accumulation of reactive oxygen species (ROS) triggers oxidative stress, activating inflammation‐related transcription factors (Ding et al. 2024). Research indicates that curcumin mitigates oxidative damage and pro‐inflammatory markers by inhibiting NF‐κB and the IκB kinase‐signaling pathway as well as reducing levels of malondialdehyde (MDA) and IL‐6 (Nunes et al. 2024; Kashyap et al. 2024). It exhibits its antioxidant effects by inhibiting superoxide radicals, hydrogen peroxide, and nitric oxide, and increasing the expression of antioxidant proteins through Nrf2 (Aggarwal et al. 2024).

Based on current and previous studies, the Cur + Pip has shown promising potential as an adjunct therapy for improving liver enzyme levels and inflammatory status, particularly in overweight adults with metabolic dysfunctions such as NAFLD and metabolic syndrome. The supplementation's overall impact on liver function and metabolic health underscores its potential as a supportive strategy for managing liver diseases and chronic inflammation (Aragón‐Vela et al. 2024; Hosseini et al. 2025).

### 4.2 Strengths, Limitations, and Future Suggestions

This meta‐analysis has several strengths, including many RCTs that enhance the reliability and generalizability of its findings. It thoroughly examines liver function enzymes and inflammatory markers, assessing the effects of Cur + Pip in adults. However, limitations include significant heterogeneity in intervention duration and participant characteristics. Furthermore, not all studies included all markers for analysis. As a result, factors like IL‐6, ALP, and TNF‐α were analyzed in 4, 4, and 5 RCTs, respectively. Future research should involve large‐scale, multicenter RCTs for longer durations.

## 5 Conclusion

The Cur + Pip appears to have a beneficial effect on AST levels, with some indication of a positive trend for ALT in certain subgroups, especially in those with overweight or metabolic dysfunction. The supplementation also reduced IL‐6, suggesting an anti‐inflammatory effect, though it did not significantly impact CRP or TNF‐α levels. Longer‐term supplementation (> 12 weeks) was particularly beneficial for AST improvement, and ALT levels, although not statistically significant, showed a more considerable decrease with extended use. Further research with larger sample sizes and longer durations could help solidify these findings, particularly in specific populations.

## Author Contributions

**Mehdi Karimi:** conceptualization (lead), data curation (lead), formal analysis (lead), funding acquisition (lead), investigation (lead), methodology (lead), project administration (lead), resources (lead), software (lead), supervision (lead), validation (lead), visualization (lead), writing – original draft (lead), writing – review and editing (lead). **Meysam Javadi:** data curation (equal), formal analysis (equal), funding acquisition (equal), investigation (equal), resources (equal), supervision (equal). **Maryam Sharifi:** conceptualization (equal), investigation (equal), methodology (equal), resources (equal), supervision (equal), validation (equal), writing – original draft (equal), writing – review and editing (equal). **Fereshteh Valizadeh:** data curation (equal), funding acquisition (equal), investigation (equal), methodology (equal), resources (equal), software (equal), supervision (equal), validation (equal), visualization (equal), writing – original draft (equal). **Mohammad Amin Karimi:** data curation (equal), project administration (equal), supervision (equal), validation (equal), writing – original draft (equal), writing – review and editing (equal). **Omid Asbaghi:** conceptualization (equal), data curation (equal), formal analysis (equal), funding acquisition (equal), investigation (equal), methodology (equal), project administration (equal), resources (equal), software (equal), supervision (equal), validation (equal), visualization (equal), writing – review and editing (equal).

## Ethics Statement

The authors have nothing to report.

## Consent

The authors have nothing to report.

## Conflicts of Interest

The authors declare no conflicts of interest.

## Supporting information


Table S1.
Table S2.
Table S3.

## Data Availability

All data generated or analyzed during this study are included in this published article.
