# Supplementary material for: Effects of Curcuminoids Plus Piperine Co‐Supplementation on Liver Enzymes and Inflammation in Adults: A GRADE‐Assessed Systematic Review and Meta‐Analysis
Source: Food Sci Nutr. 2025 Jul 14;13(7):e70588. doi: 10.1002/fsn3.70588 (PMC12257354; doi:10.1002/fsn3.70588)
Supplement: Supplementary file 1 — Table S1. Table S2. Table S3. [file FSN3-13-e70588-s001.docx]

**Supplementary Materials**

**Supplementary Table 1.** Search keywords

|  | **Query and search Keywords** |
| --- | --- |
| ***Intervention*** | “curcumin” OR “curcuma” OR “curcuminoids” OR “curcum*” OR “turmeric” OR “curcumin-piperine” |
| ***Intervention*** | “piperine” OR “pepper” OR “piper*” OR “piper longum” OR “piper nigrum” OR “co-supplementation” |
| ***Outcomes*** | “liver enzyme*” OR “hepatic enzyme*” OR “alanine aminotransferase” OR “Alanine transaminase” OR “glutamate-pyruvate transaminase” OR “GPT” OR “glutamic-pyruvic transaminase” OR “SGPT” OR “aspartate aminotransferase” OR “aspartate transaminase” OR “Glutamic-aspartic transaminase” OR “Glutamic-oxaloacetic transaminase” OR “Transaminase A” OR “alkaline phosphatase” OR “AST” OR “ALT” OR “ALP” OR “inflammatory markers” OR “inflammation” OR “C-reactive protein” OR “CRP” OR “hc-CRP” OR “hcCRP” OR “tumor necrosis factor-alpha” OR “tumor necrosis factor” OR “TNF” OR “TNF-a” OR “TNF-α” |

**Supplementary Table 2.** Search-line in databases (PubMed, ISI Web of Science, Scopus) (Title & Abstract) (Date: 1 February 2025)

| **Databases** | **Search line** | **#** |
| --- | --- | --- |
| ***PubMed*** | (("curcumin"[Title/Abstract] OR "curcuma"[Title/Abstract] OR "curcuminoids"[Title/Abstract] OR "curcum*"[Title/Abstract] OR "turmeric"[Title/Abstract] OR "curcumin-piperine"[Title/Abstract]) AND ("piperine"[Title/Abstract] OR "pepper"[Title/Abstract] OR "piper*"[Title/Abstract] OR "piper longum"[Title/Abstract] OR "piper nigrum"[Title/Abstract] OR "co-supplementation"[Title/Abstract])) AND ("liver enzyme*"[Title/Abstract] OR "hepatic enzyme*"[Title/Abstract] OR "alanine aminotransferase"[Title/Abstract] OR "Alanine transaminase"[Title/Abstract] OR "glutamate-pyruvate transaminase"[Title/Abstract] OR "GPT"[Title/Abstract] OR "glutamic-pyruvic transaminase"[Title/Abstract] OR "SGPT"[Title/Abstract] OR "aspartate aminotransferase"[Title/Abstract] OR "aspartate transaminase"[Title/Abstract] OR "Glutamic-aspartic transaminase"[Title/Abstract] OR "Glutamic-oxaloacetic transaminase"[Title/Abstract] OR "Transaminase A"[Title/Abstract] OR "alkaline phosphatase"[Title/Abstract] OR "AST"[Title/Abstract] OR "ALT"[Title/Abstract] OR "ALP"[Title/Abstract] OR "inflammatory markers"[Title/Abstract] OR "inflammation"[Title/Abstract] OR "C-reactive protein"[Title/Abstract] OR "CRP"[Title/Abstract] OR "hc-CRP"[Title/Abstract] OR "hcCRP"[Title/Abstract] OR "tumor necrosis factor-alpha"[Title/Abstract] OR "tumor necrosis factor"[Title/Abstract] OR "TNF"[Title/Abstract] OR "TNF-a"[Title/Abstract] OR "TNF-α"[Title/Abstract]) | 124 |
| ***ISI WoS*** | Results for “curcumin” OR “curcuma” OR “curcuminoids” OR “curcum*” OR “turmeric” OR “curcumin-piperine” (Topic) AND “piperine” OR “pepper” OR “piper*” OR “piper longum” OR “piper nigrum” OR “co-supplementation” (Topic) AND “liver enzyme*” OR “hepatic enzyme*” OR “alanine aminotransferase” OR “Alanine transaminase” OR “glutamate-pyruvate transaminase” OR “GPT” OR “glutamic-pyruvic transaminase” OR “SGPT” OR “aspartate aminotransferase” OR “aspartate transaminase” OR “Glutamic-aspartic transaminase” OR “Glutamic-oxaloacetic transaminase” OR “Transaminase A” OR “alkaline phosphatase” OR “AST” OR “ALT” OR “ALP” OR “inflammatory markers” OR “inflammation” OR “C-reactive protein” OR “CRP” OR “hc-CRP” OR “hcCRP” OR “tumor necrosis factor-alpha” OR “tumor necrosis factor” OR “TNF” OR “TNF-a” OR “TNF-α” (Topic) | 227 |
| ***Scopus*** | ( TITLE-ABS-KEY ( "curcumin" OR "curcuma" OR "curcuminoids" OR "curcum*" OR "turmeric" OR "curcumin-piperine" ) AND TITLE-ABS-KEY ( "piperine" OR "pepper" OR "piper*" OR "piper longum" OR "piper nigrum" OR "co-supplementation" ) AND TITLE-ABS-KEY ( "liver enzyme*" OR "hepatic enzyme*" OR "alanine aminotransferase" OR "Alanine transaminase" OR "glutamate-pyruvate transaminase" OR "GPT" OR "glutamic-pyruvic transaminase" OR "SGPT" OR "aspartate aminotransferase" OR "aspartate transaminase" OR "Glutamic-aspartic transaminase" OR "Glutamic-oxaloacetic transaminase" OR "Transaminase A" OR "alkaline phosphatase" OR "AST" OR "ALT" OR "ALP" OR "inflammatory markers" OR "inflammation" OR "C-reactive protein" OR "CRP" OR "hc-CRP" OR "hcCRP" OR "tumor necrosis factor-alpha" OR "tumor necrosis factor" OR "TNF" OR "TNF-a" OR "TNF-α" ) ) | 650 |
| ***All*** | PubMed (n=124), ISI Web of Science (n=227), Scopus (n=650)  All: 1001  Duplicates: 310 | 1001 |

**Supplementary Table 3.** Sensitivity analysis and publication bias

| **Outcomes** | **Sensitivity** | **Publication bias** |
| --- | --- | --- |
| ***CRP*** | None | *p* = 0.324 |
| ***IL6*** | Ganjali et al. (2014): (WMD: -14.75, 95% CI: -33.29, 3.78)  Rahimnia et al. (2015): (WMD: -14.64, 95% CI: -33.43, 4.14)  Miranda-Castro et al. (2022): (WMD: -0.34, 95%CI: -1.85, 1.16) | *p* = 0.132 |
| ***TNF*** | Rahimnia et al. (2015): (WMD: -1.59, 95%CI: -3.12, -0.06) | *p* = 0.498 |
| ***ALT*** | Saberi-Karimian et al. (2020): (WMD: -4.85, 95%CI: -8.97, -0.72)  Mirhafez et al. (2021): (WMD: -4.48; 95% CI: -8.68, -0.27)  Askari et al. (2022): (WMD: -5.01, 95% CI: -9.03, -0.99)  Arabnezhad et al. (2022): 95% CI -4.39 (-8.74, -0.04) | *p* = 0.601 |
| ***AST*** | Panahi et al. (2016): (WMD: -1.92, 95% CI: -4.32, 0.47)  Cicero et al. (2020): WMD: (-2.13, 95% CI: -4.55, 0.28)  Arabnezhad et al. (2022): (WMD: -2.09, 95% CI: -4.21, 0.01)  Sharrifi et al. (2023): (WMD: -1.90, 95%CI: -4.08, 0.28) | *p* = 0.606 |
| ***ALP*** | Arabnezhad et al. (2022): (WMD: -23.08, 95% CI: -37.69, -8.47) | *p* = **0.041*** |
